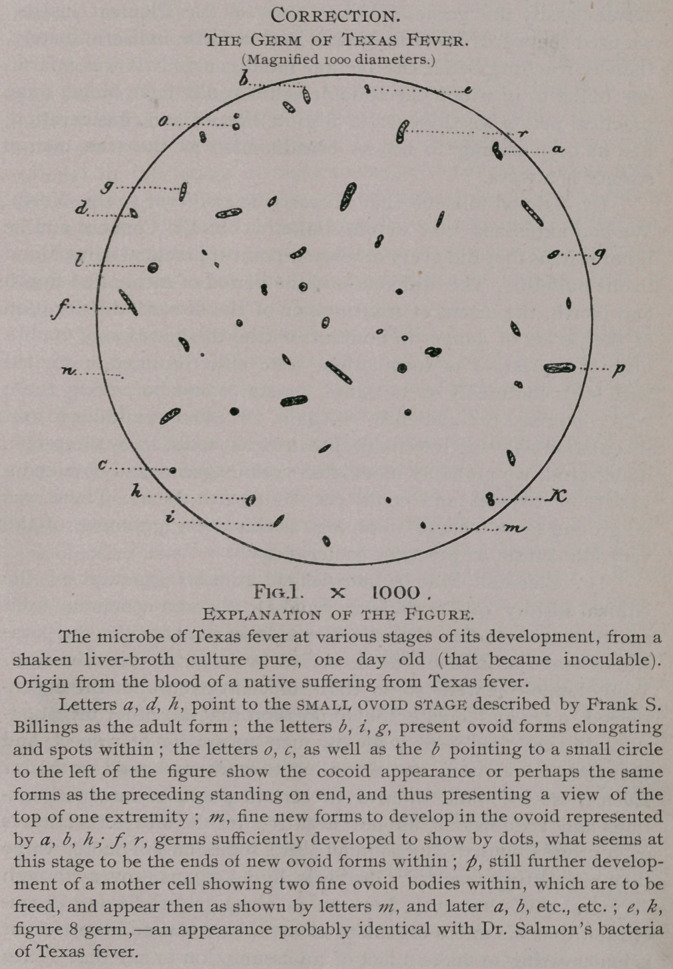# Open Letter—Texas Fever

**Published:** 1890-12

**Authors:** Paul Paquin

**Affiliations:** Office of Paul Paquin, M.D., M.V., State Veterinarian, Columbia, Mo.


### OPEN LETTER—TEXAS FEVER.

OFFICE OF PAUL PAQUIN, M.D., M.V.,
STATE VETERINARIAN,
COLUMBIA, MO., November 8th, 1890.

EDITOR JOURNAL OF COMPARATIVE MEDICINE AND VETERINARY ARCHIVES:

DEAR SIR:—In the last portion of my bulletin on Texas Fever, published in August number of the JOURNAL OF COMPARATIVE MEDICINE AND VETERINARY ARCHIVES, Fig. 1 has been so contracted, as I wrote you before, as to be absolutely useless, and then the indication under it is made to read as follows: "Fig. 1 × 2000," instead of "Fig. 1 × 1000," as it should. Please kindly correct the error.[1]

Now, I beg leave to say a few words more in regard to the criticism of Dr. Clement in our investigation of Texas fever and the discussion and paper on the same subject by Dr. Salmon. I did not wish to tire the audience by further discussion at Chicago.

I protest against the positiveness and exclusiveness with which these gentlemen explain their own opinions, and the uncalled-for allusion to jealousy which was hurled from such a high official position as Dr. Salmon's; I protest against the evident endeavor of Dr. Clement to cast a reflection on our honesty in our endeavors to fulfill our mission and duty by our people and country in investigation of Texas fever. Dr. Clement purposely or ignorantly selected the first and necessarily crude part of the first year of our investigations and compares it with the latest labors of the Bureau of Animal Industry, which had been investigating the problem for years, and tries to create the impression abroad that our work is unworthy of notice, whilst he and the gentlemen of the Bureau are impregnable. We know well the abilities of Drs. Smith, Salmon and others, and none dispute them; we give them credit and due respect. There was no occasion to write opinions in the guise of report and discuss them in such a way as to attack the professional and manly integrity of others.

[1] See next page.

Our bulletin No. 11 on Texas fever was a first report (on progress), for our people, as demanded by law, and it is explained

CORRECTION.

THE GERM OF TEXAS FEVER.

therein that it was written for the people generally, and not for scientists. We explain further that as to the germ, we do not mean to describe the biological points, and that we mean later to write on that question in particular after more complete study. We *do not* classify the germ of Texas fever as Dr. Clement insists; we used the words bacteria, germs, and so forth, indiscriminately, thereby reaching better the average intelligence. It was a farmers' bulletin, in which the amiable critic could have found some practical points worthy even of his intelligence and consideration, had he been willing to act as broadminded as the true man of science is always.

Dr. Salmon tells us that *tics* carry Texas fever to the North. We had explained that in our bulletin, but Dr. Clement and he ignored it, as they did every other new point of practical value found in our bulletin. The differences in the period of incubation South and North, the means of transmission of the disease, the duration of the period of danger in Southern cattle, the question of immunity, the question of inoculation, were either ignored or treated with such contumely as to cause general comment among those who comprised the audience. Again, Dr. Salmon alludes to our inoculation as being unreliable, because, he would have the people understand, we probably inoculated various germs and obtained a variety of diseases and could not distinguish them. There was no ground for a charge implying, as this does, ignorance of the very foundation of practical bacteriology.

Dr. Clement (who acknowledged himself ignorant of the clinical history of Texas fever), and Dr. Salmon conclude, from microscopical studies, that the Texas fever parasite and the parasite of malaria in man, as described by Leveran, are identical. Well, suppose it were so, is that sufficient excuse for these gentlemen to try and create the impression that all other investigations in that field were made by men unworthy of respect, and that their labors were not according to truth and good methods? Even if we are found in error in certain points of our investigations and conclusions, do not negative results and dire failures even prove frequently of as much, and sometimes of more, value than affirmative results? But it is by no means proven, though very probable, that even Leveran is absolutely right in his conclusion that his micro-organism is *not* a bacteria, and therefore it is endeavoring to make a fact of an assumption to say that Texas fever is *surely* not bacterial, and is identical with malaria. For years the most learned authorities thought actinomycosis was produced by a fungus, and now come Baumgarten, Bostrom, Podwissowsky, Affanassiew, Cornil, Babes, who, after deep studies, classify it among bacteria. Shall we reflect on the knowledge, honesty, integrity of Peroncito, Bollinger, Harz, Ponfic, Israël and all other scientific men who classified it among fungi?

Now, to whatever class the micro-organism of Texas fever may belong, it is not yet clear by any means—Dr. Salmon's, Dr. Smith's and Dr. Clement's opinions notwithstanding—that it is or is not of a bacterial order. In my judgment, it is more rational in this stage of our knowledge to compare Texas fever and its germ to "hemoglobinuria of cattle," described by Babes in Roumania. The blood corpuscles show about identical lesions, and Dr. Salmon's own lantern projections exposed blood corpuscles with lesions much like the illustrations of Babes. Here is what this author says:

"Bacterial hemoglobinuria of cattle . . . characterized by the condition of urine, *colored red* or *black* by *hemoglobine* . . . . and by the seat of special microbes in the *interior* of red blood corpuscles." This corresponds with the lesions of Texas fever. Babes is not exclusive, however, and does not pretend, even after long and close investigations, to say positively that the germ belongs to this or that class. He has *cultivated* it with difficulty; he inoculated it and reproduced the lesions in the blood of *small animals* and *cattle* as we had done Texas fever, and have *repeated* since the Chicago veterinary meeting. Babes further states that the microbe "appears under *various* forms." Will Dr. Clement ridicule this great author, as he tried to do with us, because we describe various forms of germs in connection with Texas fever? It is an elementary question in biology, the changeable sizes and shapes of microbes in evolution. And why not several different germs? In typhoid fever there has been a variety of germs found which are called now typical, constant and pathogenic and capable of causing this fever. This is not probable in Texas fever, but various forms may be present.

In science none can gain confidence by attempting reflections on others, no matter how humble their station, their work or their knowledge.

PAUL PAQUIN.

## Figures and Tables

**Fig.1. f1:**